# Extraction of three-dimensional shapes in glaucoma patients in response to monocular depth cues

**DOI:** 10.1007/s10384-024-01054-6

**Published:** 2024-04-10

**Authors:** Hiromasa Sawamura, Ryo Asaoka, Hiroshi Murata, Eriko Ando, Céline R. Gillebert

**Affiliations:** 1 Department of Ophthalmology, The University of Tokyo Graduate School of Medicine, 7-3-1, Hongo, Bunkyo-ku, Tokyo, 113-8655 Japan; 2 Department of Ophthalmology, Seirei Hamamatsu General Hospital, Hamamatsu, Shizuoka Japan; 3 Seirei Christopher University, Hamamatsu, Shizuoka Japan; 4 Nanovision Research Division, Research Institute of Electronics, Shizuoka University, Shizuoka, Japan; 5 The Graduate School for the Creation of New Photonics Industries, Shizuoka, Japan; 6 Department of Brain and Cognition, KU Leuven, Leuven, Belgium

**Keywords:** Glaucoma, 3D shape perception

## Abstract

**Purpose:**

To assess the impact of glaucoma on perceiving three-dimensional (3D) shapes based on monocular depth cues.

**Study design:**

Clinical observational study.

**Methods:**

Twenty glaucoma patients, subjected to binocular visual-field sensitivity (binocular-VFS) tests using a Humphrey Visual Field Analyzer, and 20 age-matched healthy volunteers, underwent two tasks: identifying the nearest vertex of a 3D shape using monocular shading (3D-SfS), texture (3D-SfT), or motion (3D-SfM) cues, and distinguishing elementary one-dimensional (1D) features of these cues. The association of the visual-field index (VFI) of binocular-VFS with 3D shape perception in glaucoma patients was also examined.

**Results:**

Glaucoma patients demonstrated reduced accuracy in distinguishing 1D luminance brightness and a larger "error-in-depth" between the perceived and actual depths for 3D-SfM and 3D-SfS compared to healthy volunteers. Six glaucoma patients with a 100% VFI for binocular-VFS exhibited a similar error-in-depth to the other fourteen glaucoma patients; they had a larger error-in-depth for 3D-SfM compared to healthy volunteers. No correlation between the error-in-depth values and the VFI values of binocular-VFS was observed.

**Conclusions:**

The 3D shape perception in glaucoma patients varies based on the depth cue's characteristics. Impaired 1D discrimination and larger thresholds for 3D-SfM in glaucoma patients with a 100% VFI for binocular-VFS indicate more pronounced perceptual deficits of lower-level elementary features for 3D-SfS and higher-level visual processing of 3D shapes for 3D-SfM. The effects of the location and degree of binocular visual-field defects on 3D shape perception remain to be elucidated. Our research provides insights into the 3D shape extraction mechanism in glaucoma.

## Introduction

Glaucoma is a leading cause of blindness worldwide [1]. It is characterized by progressive impairment of visual-field sensitivity with retinal ganglion cell loss [2, 3], resulting in decreased contrast and motion sensitivity, as well as neurodegeneration of the lateral geniculate nucleus and cortical visual system [3–7].

The binocular depth perception of individuals with confirmed or suspected glaucoma is impaired due to disruption of binocular interactions [8, 9]. Depth structure is impaired but can still be perceived; pictorial monocular depth cues, such as shading and texture, and monocular motion parallax cues contribute to this capability [10–12]. However, it is unclear whether this is the case in glaucoma patients, as little is known about the contribution of monocular depth cues to glaucoma. The impaired depth perception in glaucoma patients has been evaluated primarily in zero-order depth, which refers to the relative range or distance to the observer or fixation point, in terms of depth orders [13]. The perception of other depth derivatives, such as the deviation of a planar surface from the fronto-parallel plane (first-order depth) or depth curvature (second-order depth) [13] in glaucoma patients remains to be elucidated.

Computation of spatial depth derivatives is vital for perceiving three-dimensional (3D) shapes. These can be derived from various cues, encompassing binocular disparity and monocular depth cues such as shading, texture, and motion [13–15]. In a previous work, we studied the disparity between perceived and actual depths in 3D shape perception and determined that it was heightened in strabismus patients whose binocular stereopsis was diminished or lacking, even when the 3D shape was determined by a monocular shading cue [16]. Consequently, we theorized that 3D shape perception, delineated by monocular cues, might also be compromised in glaucoma patients due to impaired binocular depth perception. For strabismus patients, interocular suppression, mediated at the striate cortex or a higher cortical level [17–19], prevents diplopia and may influence 3D shape perception. On the other hand, in glaucoma patients, visual-field sensitivity is locally decreased rather than fully suppressed in one eye, leading to diminished visual inputs being relayed to cortical visual systems.

Our study probed how glaucoma impacts the perception of 3D shapes characterized by monocular static pictorial (shading and texture) and dynamic (motion) cues under binocular free-viewing conditions, mirroring everyday scenarios. To achieve this, we examined the disparities between the actual and perceived depths of 3D shape images [16, 20]. Visual-field defects may induce perceptual deficits in one-dimensional (1D) elementary visual features that act as cues for 3D shape perception. Thus, to assess perception of these foundational features, we employed two tasks: 3D shape perception and 1D elementary feature discrimination. Additionally, the binocular visual-field sensitivity (binocular-VFS) of glaucoma patients was measured to determine its influence on 3D shape perception.

## Patients and methods

### Study design and participants

This cross-sectional, observational study adhered to the STROBE cross-sectional reporting guidelines [21]. The study protocol was approved by the Ethics Committee of Tokyo University Hospital. All participants provided written informed consent in accordance with the Declaration of Helsinki.

Between November 2018 and February 2020, 20 patients with glaucoma (mean ± SD age: 56.7 ± 13.6 years; 7 men and 13 women) were enrolled; in addition, 20 age-matched healthy volunteers (mean ± SD age: 56.0 ± 9.6 years, 10 men and 10 women) were enrolled as normal controls. The eligibility criteria for the glaucoma patients included a diagnosis of normal-tension glaucoma, receiving treatment at Tokyo University Hospital for > 1 year with topical agents or surgical intervention, experiencing a visual-field scotoma in the affected eye(s), and undergoing medical check-ups every 3 months.

### Behavioral procedures

The visual stimuli and tasks performed in the present study have been described previously [16]. Briefly, the tasks investigated 3D shape perception and discrimination of 1D elementary features that serve as cues for 3D shape perception. The stimuli were presented on a 13-in LCD monitor (resolution: 1,920 × 1,080 pixels; refresh rate: 60 Hz) with the participants seated 40 cm away from the screen, like our previous study, and 30 cm from the Humphrey Field Analyzer (HFA). The head of each participant was constrained using a head and chin rest. Stimulus presentation and response registration were controlled by a personal computer using in-house software and Presentation 11.3 (Neurobehavioral Systems).

#### Task 1: Three-dimensional shape perception 

**Fig. 1:**
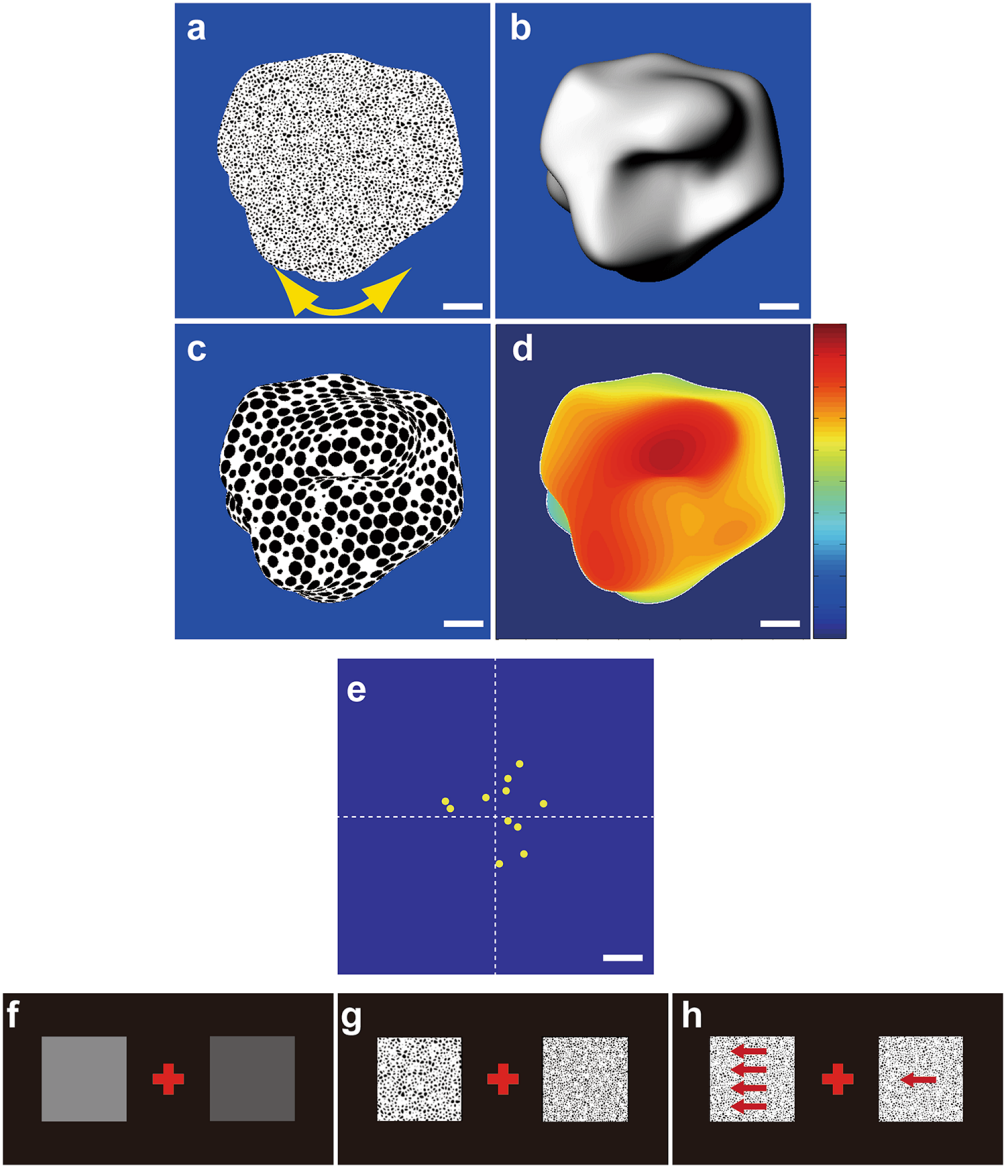
Display of a visual stimulus: **a** 3D shape defined by motion (3D-SfM), **b** shading (3D-SfS), and **c** texture (3D-SfT). **d** Depth–color map of the stimulus, with the depth difference (cm) from the base of the visual stimulus (highlighted with a blue background) represented by the color bar (blue, 0 cm; red, 5 cm). The white scale bar is 64 pixels. **e** The x-y distribution of the true global maximum for each stimulus (marked by yellow dots) is set against the blue backdrop of the visual stimulus. A white dotted line divides the four quadrants. The white scale bar is 64 pixels. **f**–**h** Elementary feature discrimination tasks for brightness of luminance, texture coarseness, and speed of motion, respectively

Visual stimuli employed in previous studies, created, and rendered in three modalities (shading, 3D-SfS; texture, 3D-SfT; motion, 3D-SfM), were used [16, 20]. These stimuli depicted 11 randomly generated complex and meaningless 3D objects including a variety of hills, ridges, valleys, and dimples [22–25]. Examples of a visual stimulus described under each modality are shown in Fig. 1a–c. A depth–color map of the stimulus is shown in Fig. 1d. Stimuli from each of the three modalities were presented in blocks of 11 trials, in which each 3D shape was shown once per block in a random order. In each trial, a single 3D shape (average size: 9° × 9°) was presented at the center of the screen. The participants were instructed to identify the foremost vertex on the convexity of the 3D surfaces (the highest convex point or global maximum of the surface, i.e., the point nearest to the observer) by superimposing a red cross on this point using a computer mouse under free-viewing and binocular conditions without any time limitation; these conditions were similar to those in daily life. Eye-tracking was not performed. The locations of the true global maximum of the 11 visual stimuli are shown in Fig. 1e. The depth difference between the global maximum of the surface, as identified by the participant, and the true global maximum was defined as the “error-in-depth” (cm).

#### Task 2: simple feature discrimination

Two squares (5.7° × 5.7°) were presented simultaneously on the monitor, for 6 s, on either side (at 6.4°) of the fixation point, as described previously. Each square consisted of a single luminance value (Fig. 1f), texture coarseness (Fig. 1g), or speed of motion (Fig. 1h). Three levels of each cue were presented: a basic speed (1.9 °/s) and 20% faster or slower speeds, a basic luminance (60% greyscale) and 15% brighter or darker luminances, a basic texture (Fig. 1g, left stimuli) and 20% finer or 20% coarser textures. For each cue, three combinations of squares were derived from the different stimulus levels, and each combination was presented four times, yielding 12 trials. Moreover, an identical stimulus level was presented in the two squares in three trials. Thus, 15 trials for each cue were presented in random order and a block design was used. The participants selected the square with the faster motion, brighter luminance, or coarser texture by pressing the right or left shift key on the keyboard. If the two squares were considered identical, the participant was required to press the space key.

### Visual-field sensitivity testing

Patients underwent visual-field testing in each eye using the HFA 24-2 or 30-2 Swedish Interactive Threshold Algorithm (SITA) standard program semi-annually. Details on visual acuity, foveal sensitivity (dB), mean deviation value (dB), pattern standard deviation value (dB), and superior and inferior total deviation values (dB) captured using the HFA are summarized in Table 1. According to the glaucoma staging system of Mills et al., 2006, who modified the Hodapp-Parrish-Anderson criteria [26], eight, two, seven, and three participants were classified as stages 1, 2, 3, and 4, respectively, based on their visual-field scores for their worst eye [27]. The binocular visual fields of the glaucoma patients were assessed to gauge the influence of binocular-VFS on 3D shape perception. Due to the lack of available appropriate instruments to evaluate binocular-VFS, we used the method delineated by Matsuura et al. [28]. Briefly, the glaucoma patients were tested using the right 30-2 standard SITA program, but with both eyes open. During this binocular visual-field testing, the position of the chinrest was adjusted to its furthest left setting, allowing patients to position their chins over the right side of the chinrest. This configuration facilitated patients in aligning with the perimeter by adjusting both the vertical position of their head and the horizontal alignment relative to the bridge of their nose.

**Table 1:** Characteristics of visual acuity and index of visual field

Participants	VA	MD(dB)	PSD(dB)	msTD (dB)	miTD (dB)
	Right	Right	Right	Right	Right
	Left	Left	Left	Left	Left
1	20/20	− 9.81	14.24	− 2.84	− 14.30
	20/20	− 9.11	11.75	− 4.35	− 9.97
2	20/20	− 8.39	16.56	− 16.86	1.32
	20/20	− 4.88	9.63	− 7.00	− 1.73
3	20/20	− 17.29	14.37	− 25.94	− 11.70
	20/20	− 13.30	11.42	− 19.95	− 10.81
4	20/25	− 24.70	11.35	− 17.89	− 29.11
	20/20	− 3.43	4.04	− 4.83	− 2.38
5	20/20	− 2.59	4.72	− 4.41	− 1.08
	20/20	− 1.66	1.62	− 0.97	− 1.92
6	20/20	− 11.43	13.24	− 20.62	− 3.85
	20/20	− 10.99	10.97	− 19.96	− 3.69
7	20/20	− 11.05	13.26	− 20.46	− 2.58
	20/40	− 25.98	7.72	− 29.58	− 23.73
8	20/25	− 11.49	11.99	− 7.76	− 13.57
	20/40	− 7.49	9.83	− 4.32	− 6.70
9	20/20	− 0.45	4.04	− 1.65	1.38
	20/20	2.53	1.00	2.69	2.50
10	20/20	− 0.90	1.35	− 0.31	− 1.35
	20/20	− 3.91	5.16	− 6.11	− 2.31

Participants	VA	MD(dB)	PSD(dB)	msTD (dB)	miTD (dB)
	Right	Right	Right	Right	Right
	Left	Left	Left	Left	Left
11	20/20	− 5.37	7.24	− 5.00	− 7.34
	20/20	− 10.37	11.27	− 15.27	− 5.42
12	20/20	− 25.81	12.58	− 28.35	− 21.11
	20/20	− 16.77	16.08	− 4.08	− 26.23
13	20/20	− 8.36	11.80	− 17.83	− 3.00
	20/20	− 6.79	5.65	− 8.84	− 5.27
14	20/20	− 17.93	12.22	− 18.62	− 15.23
	20/20	− 15.33	15.59	− 29.42	− 3.00
15	20/20	− 1.54	6.63	− 1.43	− 1.78
	20/20	− 4.94	10.79	− 11.95	− 0.84
16	20/20	− 1.74	2.49	− 0.62	− 2.88
	20/20	− 19.20	14.71	− 17.65	− 17.81
17	20/20	− 3.54	3.73	− 3.11	− 4.54
	20/20	− 1.57	4.17	− 3.27	− 0.88
18	20/20	− 1.92	2.85	− 2.43	− 0.81
	20/20	-5.21	10.13	− 10.89	− 0.89
19	20/20	− 4.43	12.34	− 13.34	2.46
	20/20	1.13	1.39	1.38	1.15
20	20/20	1.43	1.88	2.50	0.38
	20/20	− 3.19	7.28	1.73	− 7.46

*VA* visual acuity, *MD* mean deviation, *PSD* pattern standard deviation, *mTDsup* mTDinf mean total deviation in superior or inferior visual hemifield

### Statistical analysis

The accuracy for each cue in the 1D feature discrimination task, together with the error-in-depth values for each cue in the 3D shape perception task, were compared between the glaucoma patients and the healthy volunteers using Welch’s t-test. In the formula (*t*[df] = X, p = Y), *t*, df, and p indicate the t-value, degree of freedom, and p-value, respectively. The threshold for statistical significance was set at p < 0.05, with the adjusted threshold determined at p < 0.017 after applying Bonferroni correction.

As depicted in Fig. 1e, the positions of the true global maximum of the 3D visual stimuli within the visual field were evenly distributed around the center of the stimuli. The perception of 3D shape, as examined in this study, necessitates integration over a retinal area that surpasses single tested points in the HFA. Additionally, the specific region of the visual field responsible for 3D shape perception remained ambiguous due to the binocularly free-viewing conditions. The diminished monocular visual-field sensitivity in glaucoma patients manifested across various regions of the visual field because of the sporadic nature of visual-field defects. In a report that assessed temporal contrast sensitivity in the parvocellular and magnocellular pathways among healthy subjects, in subjects suspected of glaucoma and perimetric glaucoma patients, the stages of glaucoma were controlled [29]; whereas in the current study the glaucoma patients were considered together despite their wide range of visual-field defects and glaucoma stages [27]. Therefore, to evaluate the influence of binocular-VFS on 3D shape perception, we used visual-field index (VFI) values [30] of binocular-VFS. The VFI has more relevance to the central visual field, which largely covered the 3D-shape images. The correlations between VFI values (%) of binocular-VFS and error-in-depth values (cm) were evaluated using Spearman’s rank test. The glaucoma patients were divided into two subgroups based on their VFI values of binocular-VFS. The VFI values of binocular-VFS range from 0% (perimetrically blind) to 100% (normal visual field) [30]; patients with a VFI of 100% were classified as unimpaired, whereas all other patients were classified as impaired. The error-in-depth values of the healthy volunteers and the two subgroups were scrutinized using analysis of variance (ANOVA), taking the three groups as factors, and adjusting for significance using Bonferroni correction.

## Results

**Fig. 2:**
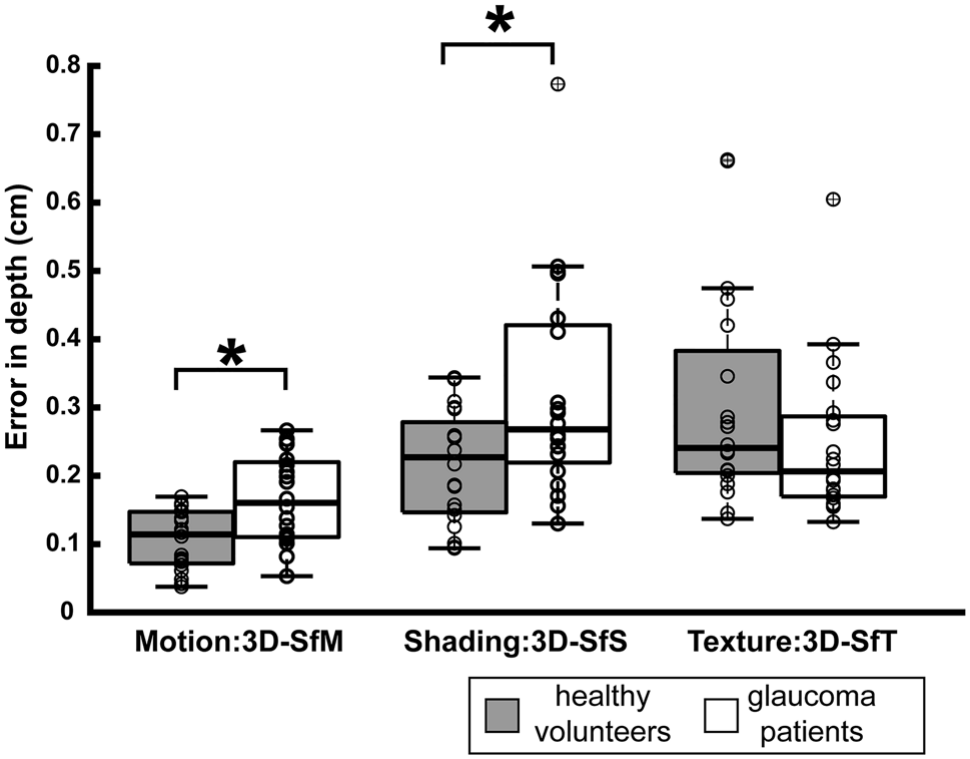
Box plots represent the error-in-depth (cm) during the perception of 3D shapes derived from motion, shading, and texture cues. Gray and white boxes display results for the healthy volunteers and glaucoma patients, respectively. The bold black line within a box represents the median, while the top and bottom edges of a box indicate the 75^th^ and 25^th^ percentiles, respectively. Crosses mark outliers, and the whiskers reach the furthest data points, excluding outliers. White dots showcase individual participants’ responses. Asterisks signify statistical significance (*p* < 0.017)

Comparisons of the error-in-depth values between the glaucoma patients and the healthy volunteers are shown in Fig. 2. Welch’s t-test revealed that glaucoma patients exhibited larger error-in-depth values than healthy volunteers for 3D-SfM (*t*[33.22] = 3.43, p = 0.001) and 3D-SfS (*t*[28.52] = 2.62, p = 0.014). For 3D-SfT, no significant discrepancies were observed between the two groups (*t*[34.71] = 1.35, p = 0.18).

Discrepancies in 3D-SfM and 3D-SfS results between the glaucoma patients and the healthy volunteers were noted. This implied that whenever these differences stemmed primarily from diminished visual inputs at the retinae in glaucoma patients, the thresholds for the unimpaired subgroup would approximate those of the healthy volunteers. Conversely, whenever retinal visual input exerted a minimal influence on these differences, the unimpaired subgroup thresholds would align more closely with the impaired subgroup. This hypothesis was tested. Out of the 20 glaucoma patients, 6 met the criteria for the unimpaired subgroup (cf. Patients and Methods). The error-in-depth (mean ± SD) values of each group, namely the healthy volunteers, unimpaired subgroup, and impaired subgroup, are tabulated in Table 2. The threshold of the unimpaired subgroup was similar to that of the impaired subgroup for both 3D-SfM and 3D-SfS. Significant variance in the mean thresholds of error-in-depth values was observed among the three groups for 3D-SfM at the corrected level and for 3D-SfS at the uncorrected level, with an adjusted p-value set at 0.025 (Table 2).

**Table 2:** Error-in-depth (mean± SD, cm) and the statistics for 3D-SfM and 3D-SfS in each group: heathy volunteers and subgroups of glaucoma patients: unimpaired (100% of VFI) and impaired (< 100 % of VFI) subgroups

Stimuli	Healthy	Unimpaired	Impaired	df	F	*p*
(*N *= 20)		(*N* = 6)		(*N* = 14)		
3D-SfM	0.106 ± 0.043	0.167 ± 0.064	0.164 ± 0.067	[2, 36]	5.743	0.007*
3D-SfS	0.214 ± 0.082	0.316 ± 0.120	0.319 ± 0.175	[2, 36]	3.344	0.046

*N* number of subjects

3D-SfM, 3D-SfS: 3D shape from monocular motion (3D-SfM) or shading (3D-SfS) cues

Healthy, Umimpaired, Impaired : healthy volunteers, unimpaired or impaired subgroup of glaucoma

df, F, p: degree of freedom, F-value and p-value obtained from ANOVA

^*^indicates statistical significance at corrected level (p < 0.025)

The correlation coefficients (ρ) between the VFI values of binocular-VFS and the error-in-depth values of patients with glaucoma were 0.0053 for 3D-SfM, -0.079 for 3D-SfS, and -0.086 for 3D-SfT (all p > 0.7).

**Fig. 3:**
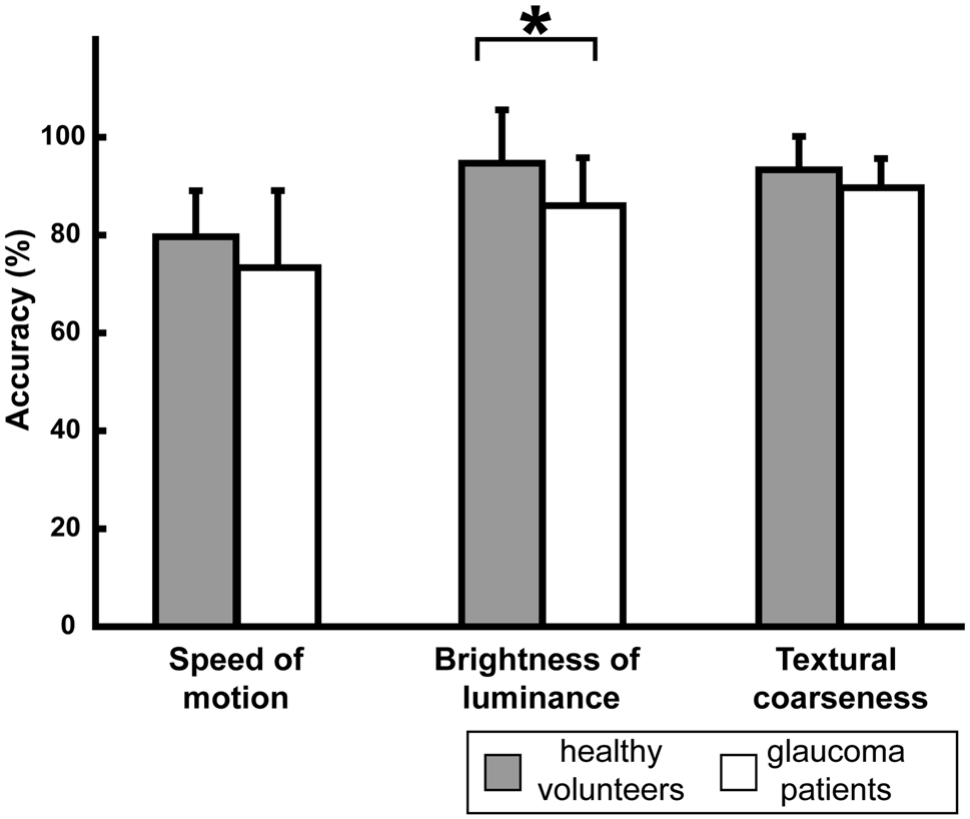
A comparison of accuracy in the feature discrimination task between healthy volunteers (represented by gray bars) and glaucoma patients (white bars) across different cues: speed of motion, brightness of luminance, and textural coarseness. The error bars denote SE. Statistically significant variations are marked with * (*p* < 0.017)

The accuracy for the feature discrimination task is shown in Fig. 3. For patients with glaucoma, accuracy in distinguishing the brightness of luminance was significantly lower (*t*[32.15] = 3.48, p = 0.001) but not for speed of motion (*t*[33.84] = 1.48, p = 0.148) or textural coarseness (*t*[33.98] = 1.373, p = 0.179).

## Discussion

We determined that glaucoma correlated with higher error-in-depth values, which represent the depth variance between the true global maximum and the participant-identified global maximum of the curved 3D surface, a pivotal element in 3D shape perception. Such disparities were observed when 3D shapes were delineated by motion and shading monocular depth cues, but not when they were delineated by the texture cue. Intriguingly, even among glaucoma patients with a VFI value of 100% for binocular-VFS, the error-in-depth values increased.

For glaucoma patients, the accuracy in distinguishing the brightness of luminance was noticeably lower, possibly due to the diminished contrast sensitivity arising from progressive dysfunction or loss of retinal ganglion cells in glaucoma [31–33]. The prevalent notion, supported by numerous reports, is that glaucoma primarily impairs contrast sensitivity [34–36]. Conversely, the accuracy in identifying the speed of motion and textural coarseness remained largely consistent between the glaucoma patients and healthy volunteers. This implies that any disparities between the two groups in error-in-depth values for 3D shapes defined by motion and texture cues were barely influenced by perceptual deficits in the rudimentary visual representations of these elementary features. While the threshold of motion-sensitivity is reported to be lower in glaucoma patients [31, 37, 38], this does not accord with our findings. One explanation could be that our approach to evaluating visual processing differed from other studies, primarily due to the limited levels of stimulus we employed. However, with only three stimulus levels significant discrepancies in luminance discrimination were still observed, indicating their sufficiency.

Glaucoma’s impact on 3D shape perception is intrinsically linked to the 3D shape’s characteristics. Compared to healthy participants, glaucoma patients exhibited greater error-in-depth values for 3D-SfM and 3D-SfS, but not for 3D-SfT. This implies that the thresholds for monocular motion and shading depth cues might increase due to glaucoma. One perspective suggests that perceptual deficits in elementary visual features could influence 3D shape perception, a view that seems more applicable to 3D-SfS, given the pronounced inaccuracies in luminance discrimination. Another plausible hypothesis pertains to the discontinuity of the specular reflection cue in glaucoma patients concerning 3D-SfS. As the specular reflections in 3D-SfS relay crucial data about surface orientation and finish [24, 25, 39–41], a disrupted specular reflection cue may reduce the inputs to the cortical visual pathway for 3D-SfS perception, resulting in an augmented error-in-depth values.

The variations in 3D shape perception based on cues hint at the involvement of advanced cortical processing. Glaucomatous visual-field defects may differentially impact 3D shape perception delineated by monocular depth cues due to distinct cortical processing [13]. Numerous imaging studies indicate differential involvement of ventral and dorsal visual pathways in 3D shape perception, contingent upon the defining cue, in the dorsal visual pathway for 3D-SfM, temporal regions of the ventral visual pathway for 3D-SfS, and ventral and dorsal visual pathways for 3D-SfT [20, 22, 42]. Neurodegeneration observed in the cortical visual system of glaucoma patients corroborates this theory [7, 43]. Despite indiscriminate damage to ganglion cells in both the parvocellular and magnocellular pathways of glaucoma patients [7, 44], functional connectivity diminishes primarily in the dorsal visual pathway [45]. It is plausible that their 3D-SfM perception is influenced more by higher-level cortical processing, whereas their 3D-SfS perception is more influenced by perceptual deficits in elementary visual features.

Our subgroup analysis revealed that the error-in-depth values for 3D-SfM in glaucoma patients were not dependent on their VFI values of binocular-VFS but were significantly larger than in healthy volunteers. This finding also supports the view of a large contribution of higher-level cortical processing to 3D-SfM in glaucoma, although the unimpaired subgroup may have had reduced visual-field sensitivity, especially in the peripheral visual field [30], resulting in less visual input that would in turn have affected their 3D-SfM perception.

It was hypothesized that larger VFI values for binocular-VFS would be associated with smaller error-in-depth values in glaucoma patients, but no such correlation was observed for 3D-SfS, 3D-SfM, or 3D-SfT. This was probably attributable to the wide variety of visual-field defects among our glaucoma patients (Table 1). To complete the task used in this study, the patients had to distinguish the 3D shapes from the background, identify the overall 3D shapes, and identify the foremost vertex of the overall shape, rather than the local 3D structure [46]. The peripheral visual field probably provided a frame of reference for the first step, and the central visual field probably facilitated the second and third steps. Indeed, 3D shape perception in the peripheral visual field has been reported [46]. Therefore, reduced visual inputs in both the central visual field and the peripheral visual field may affect 3D shape perception in glaucoma. Further work is needed to reveal the effects of the location and degree of visual-field defects on the 3D shape perception in glaucoma patients, while controlling for glaucoma stage [27] and the location of visual-field defects.

The 3D shapes chosen for this study were considered sufficient to determine the threshold of 3D shape perception based on a previous study of patients with posterior cortical atrophy [20]. The visual stimuli were designed to reveal possible association between impaired 3D shape perception and volume loss in the cortex, based on previous psychophysical and functional magnetic resonance imaging studies [13, 22, 39]. The study revealed increased error-in-depth values for 3D-SfS, 3D-SfM and 3D-SfT in a depth cue-independent manner [20], in contrast to a depth cue-dependent manner in patients with glaucoma. The difference can be explained by the primary diseases, posterior cortical atrophy, or glaucoma, where the cortex or retinal ganglion cells are mainly damaged, respectively [2, 3, 47]. The same visual stimuli and task procedures were also used to test patients with strabismus, and increased error-in-depth values for 3D-SfS were associated with a lack of binocular stereopsis [16]. Therefore, the larger thresholds observed for 3D-SfS in this study could be attributed to impaired binocular depth perception, which is also observed in glaucoma [8, 9]. However, compared with the strabismus patients, those with glaucoma were less able to distinguish the brightness of luminance. Therefore, the mechanism that underlies error-in-depth in relation to 3D shape perception may differ between the two groups. Difficulty in perceiving elementary features of 3D shapes because of reduced retinal inputs may be important, as discussed above.

This study had some limitations. First, regarding the threshold of the feature discrimination tasks for evaluating perceptual deficits in low-level visual representations of the elementary features, we did not employ the QUEST or adaptive staircase procedures, which allow precise estimation of the threshold [48]. Second, 3D shape perception was assessed in a relatively small number of glaucoma patients. We cannot exclude that the absence of significant differences between the glaucoma patients and the healthy volunteers in tasks 1 and 2 are due to a lack of power. Third, depth information at a single point was used to evaluate 3D shape perception; judgments of relative depths at two local points on a visual stimulus or the surfaces of a 3D shape at different locations were not evaluated [23, 39].

In conclusion, glaucoma coarsened the perception of 3D shapes in a depth cue-dependent manner. It is plausible that reduced visual input caused by glaucoma resulted in perceptual deficits in low-level visual representations of elementary features and may have affected distinct 3D shape processing in the extrastriate cortex that depends on 3D-shapes’ characteristics. Both mechanisms appear to contribute to the impairment in 3D shape perception in glaucoma.
